# Dental Chemistry and Metallurgy

**Published:** 1896-04

**Authors:** 


Dental Chemistry and Metallurgy. Fourth Edition, revised and enlarged, with many illustrations. By Clifford Mitchell, A.M., M.D.

The popularity and value of this work is shown by the call for the successive editions. The work has been much enlarged, more than one hundred new pages have been added embracing some very important matter, among which is Physiological Chemistry as it pertains to digestion. Much attention is also given in this edition to the germ theory, ptomains, lucomains and various toxins, and considerable matter in the way of experimental chemistry, both organic and inorganic, has been added.

The work is well adapted for dental college classes and especially for those not connected with medical colleges or universities. The work is eminently practical throughout and will be of great value to the practitioner as well as the student. It should be found in the library of every dentist. It is published by the W. T. Keener Co., of Chicago, and is well gotten up in every respect.

